# Supplementary material for: Field to Greenhouse: How Stable Is the Soil Microbiome after Removal from the Field?
Source: Microorganisms. 2024 Jan 5;12(1):110. doi: 10.3390/microorganisms12010110 (PMC10818785; doi:10.3390/microorganisms12010110)
Supplement: Supplementary file 1 [file microorganisms-12-00110-s001.zip › microorganisms-2788548-supplementary.pdf]

Supplementary Information

# Field to Greenhouse: How Stable Is the Soil Microbiome after Removal from the Field?

**Priyanka Kushwaha [1], Ana L. Soto Velázquez [1], Colleen McMahan [2] and Julia W. Neilson [1,*]**

[1] Department of Environmental Science, The University of Arizona, Tucson, AZ 85721, USA; pkushwaha@arizona.edu (P.K.); anasotovelazquez@catworks.arizona.edu (A.L.S.V.)
[2] USDA Agricultural Research Service, Western Regional Research Center, Albany, CA 94710, USA; colleen.mcmahan@usda.gov
* Correspondence: jneilson@arizona.edu

**Table S1:** Soil moisture content measurements for the five bins during the 9-weeks of soil storage at 3°C. The average gravimetric moisture content of field soils on the sampling day was 8.1% and the bins were approximately maintained to that level. Letter superscripts a, b, c, and d correspond to the soil microbiome analyses time points, week 0 (starting point), week 3, week 6, and week 9, respectively.

| Timeline | Moisture Content (%) | | | | |
|---|---|---|---|---|---|
| | Bin 1 | Bin 2 | Bin 3 | Bin 4 | Bin 5 |
| Week 0[a] | 7.9 | 7.9 | 8.1 | 7.8 | 7.9 |
| Week 1 | 7.6 | 7.6 | 7.7 | 7.4 | 7.7 |
| Week 2 | 7.9 | 8.4 | 8.4 | 7.7 | 7.8 |
| Week 3[b] | 7.6 | 8.2 | 8.3 | 7.7 | 7.5 |
| Week 4 | 7.8 | 8.1 | 8.0 | 7.5 | 7.4 |
| Week 5 | 8.4 | 9.0 | 9.0 | 8.5 | 9.0 |
| Week 6[c] | 8.3 | 8.6 | 9.1 | 8.7 | 8.7 |
| Week 7 | 8.2 | 8.5 | 8.8 | 8.2 | 7.9 |
| Week 8 | 8.5 | 8.6 | 8.6 | 7.8 | 8.4 |
| Week 9[d] | 8.5 | 8.5 | 8.3 | 8.4 | 8.2 |

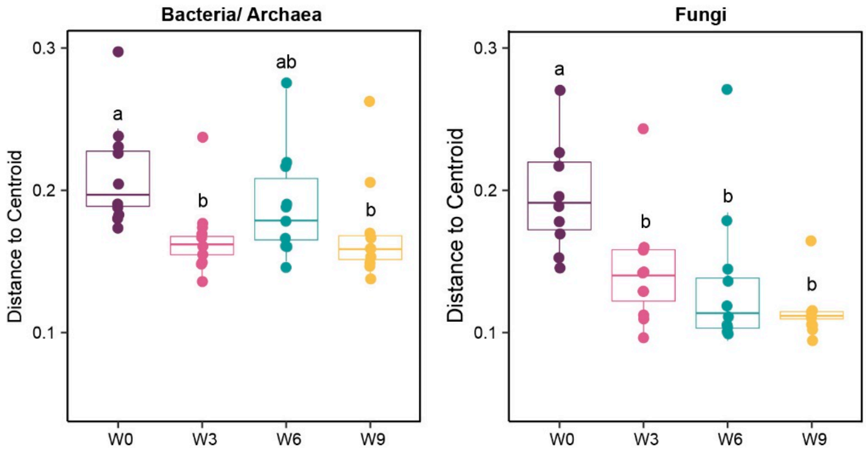


**Figure S1:** Average distance to centroid of the microbial community across the time points. Community composition beta-dispersion was calculated across the time-points using Bray-Curtis distance. Bacterial/archaeal and fungal community dispersal within the time-points varied significantly between time points which is represented by the different letters (Kruskal-Wallis test; $p \leq 0.05$). W0; Week 0, W3; Week 3, W6; Week 6, and W9; Week 9.
